# PDZK1 Protects Against RPE Senescence by Targeting the 14‐3‐3ε‐mTOR Axis to Attenuate Early Diabetic Retinopathy

**DOI:** 10.1002/advs.202511288

**Published:** 2025-08-25

**Authors:** Jian Zhao, Junbiao Zhang, Yanli Liu, Lili Wang, Chunxi Huang, Wei Chi, Meixia An

**Affiliations:** ^1^ Department of Ophthalmology The Third Affiliated Hospital Southern Medical University Guangzhou 510630 China; ^2^ The Third School of Clinical Medicine Southern Medical University Guangzhou 510630 China; ^3^ Guangdong Provincial Key Laboratory of Bone and Joint Degeneration Diseases Guangzhou 510630 China; ^4^ Shenzhen Eye Hospital Shenzhen Eye Center Southern Medical University Shenzhen 518040 China

**Keywords:** cell senescence, diabetic retinopathy, PDZK1, 14‐3‐3ε, retinal pigment epithelium

## Abstract

Diabetic retinopathy (DR) is the leading cause of blindness among working‐age adults, yet its pathogenesis remains incompletely understood. The retinal pigment epithelium (RPE) plays a vital role in maintaining retinal homeostasis. In this study, the expression of senescence marker protein p16 is observed to be upregulated in the RPE of early DR mouse models. Transcriptomic profiling reveals that PDZ domain protein 1 (PDZK1) expression is downregulated in RPE cells after 48 hours of high‐glucose stimulation. Overexpression of PDZK1 reduces senescence markers in RPE cells, promoting cell proliferation and transport functions. Mechanistically, PDZK1 alleviates RPE cell senescence by interacting with 14‐3‐3ε to regulate the mTOR pathway, which is closely related to reducing oxidative stress and enhancing autophagy flux. In streptozotocin‐induced DR mouse models, both PDZK1 overexpression‐mediated senescence inhibition and Nutlin‐3a‐induced clearance of senescent RPE cells successfully downregulate retinal senescence markers and improve early‐stage DR lesions. In summary, this study identifies a novel PDZK1‐14‐3‐3ε‐mTOR axis governing high‐glucose‐induced RPE cell senescence, and provides the first direct evidence linking RPE cell senescence to DR pathogenesis. These findings reveal a promising therapeutic strategy for DR intervention.

## Graphical abstract

PDZK1 down‐regulation couples high‐glucose stress to RPE senescence via a PDZK1–14‐3‐3ε–mTOR axis. Restoring PDZK1 or clearing senescent cells rescues retinal homeostasis and mitigates early diabetic retinopathy (DR). This study provides direct evidence linking RPE cell senescence to DR pathogenesis and reveals a promising therapeutic strategy for DR intervention.

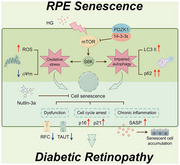

## 1 Introduction

Diabetic retinopathy (DR) is one of the most serious complications of diabetes mellitus (DM) and has become the main cause of blindness among working‐age people worldwide.^[^ ^1^ ^]^ Current research on DR mainly focuses on the neurovascular unit. However, the pathogenesis of DR is extremely complex and has not yet been fully elucidated. The retinal pigment epithelium (RPE) is a highly differentiated monolayer of pigment cells that plays a vital role in transporting choroidal nutrients and maintaining retinal metabolism.^[^ ^2^ ^]^ Therefore, recent views suggest that the important role of RPE in the development of DR may have been overlooked.^[^ ^3^ ^]^ Retinal laser photocoagulation is an effective measure to stabilize DR, and some believe that one of its main mechanisms of action is to improve the oxygen supply from the choroid.^[^ ^4^ ^]^ Our previous study also suggests that preserving RPE functional integrity to maintain choroidal circulation support for the retina represents a promising therapeutic strategy for DR.^[^ ^5^ ^]^

Stress‐induced premature senescence (SIPS) induced by various stressors is an irreversible cell cycle arrest state.^[^ ^6^ ^]^ The accumulation of senescent cells can affect organ or tissue function and increase the risk of diseases such as DM.^[^ ^7^ ^]^ For example, senescent cells can propagate senescence to neighboring cells and trigger pathological angiogenesis by expressing and releasing senescence‐associated secretory phenotype (SASP).^[^ ^8^ ^]^ A recent study found that the expression of the senescence marker protein p16 was significantly increased in the RPE of DR patients.^[^ ^9^ ^]^ However, the direct impact of RPE cell senescence on DR has not been studied, and its mechanism is even less clear.

In our previous research, we observed decreased expression of PDZ Domain Containing 1 (PDZK1) in the serum of DR patients compared to healthy subjects.^[^ ^10^ ^]^ PDZK1 is a PDZ domain‐containing membrane scaffold protein widely expressed in a variety of epithelial cells.^[^ ^11^ ^]^ It plays a key role in cell signal transduction and ion channel regulation by interacting with a variety of ion channels, transporters, and receptors. For example, PDZK1 can regulate the absorption of carnitine by the organic cation transporter (OCTN) in the intestine, regulate the transport of glycylsarcosine by the oligopeptide transporter (PEPT2), and participate in the regulation of the urate/anion exchanger (URAT1) in renal tubular uric acid reabsorption.^[^ ^12^, ^13^, ^14^ ^]^ Notably, a recent study has shown that PDZK1 loss promotes chondrocyte senescence in osteoarthritis.^[^ ^15^ ^]^ However, the role of PDZK1 in DR pathogenesis remains to be further elucidated.

The mammalian target of rapamycin (mTOR) serves as a critical regulator of protein translation in senescent cells.^[^ ^16^, ^17^ ^]^ The mTOR‐S6 kinase (S6K) pathway not only regulates cell proliferation and metabolic responses, but is closely related to oxidative stress and autophagy.^[^ ^18^, ^19^, ^20^ ^]^ Furthermore, this pathway is also involved in various pathological processes, including aging, DM, and DR.^[^ ^21^ ^]^ As a crucial subtype of the 14‐3‐3 protein family, 14‐3‐3ε interacts with the mTOR pathway and facilitates mRNA translation and protein synthesis through S6K activation, thereby exerting essential functions in fundamental biological processes such as cellular signal transduction, cell cycle progression, and metabolic regulation.^[^ ^22^, ^23^ ^]^

In this study, we selected streptozotocin (STZ)‐induced diabetic mice as an early DR animal model to confirm that DM can induce RPE cell senescence. Our findings demonstrate that high glucose conditions significantly downregulate PDZK1 expression in RPE cells. Using high‐glucose‐stimulated RPE cell models, we further established that PDZK1 protects against RPE cell senescence and transport dysfunction by modulating the 14‐3‐3‐mTOR pathway, a process mechanistically linked to oxidative stress and autophagy impairment. Nutlin‐3a has been confirmed as a senolytic agent that can target and eliminate senescent RPE cells.^[^ ^24^ ^]^ Importantly, we identified that therapeutic interventions using either PDZK1‐overexpressing lentivirus or Nutlin‐3a via intravitreal injection in STZ‐induced diabetic mice effectively attenuated early DR pathological changes. These findings provide novel insights into DR pathogenesis and suggest promising therapeutic strategies targeting RPE senescence.

## 2 Results

### 2.1 High Glucose Induced RPE Cell Senescence and Decreased PDZK1 Expression

**Figure 1:**
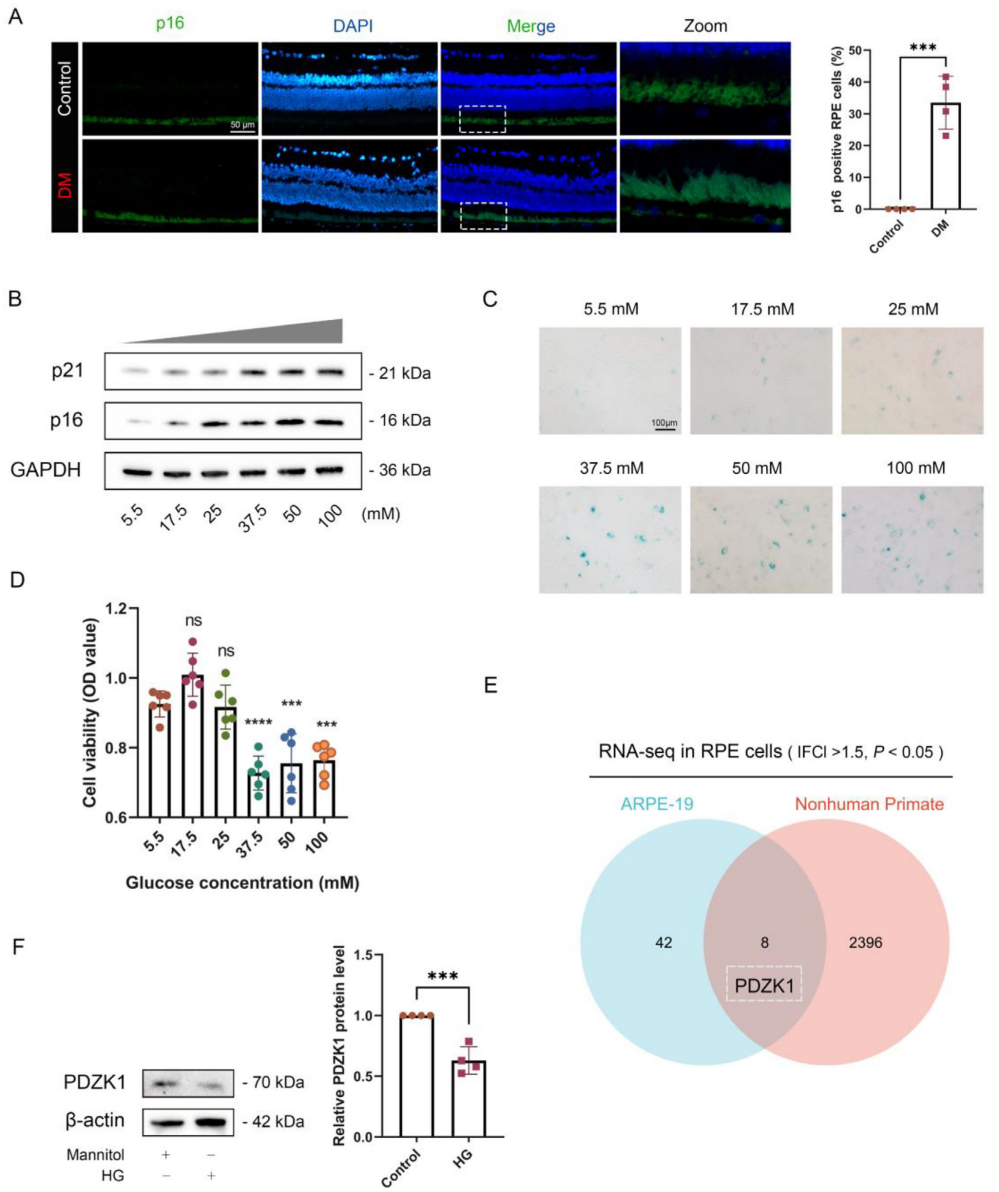
High glucose induced RPE cell senescence and decreased PDZK1 expression. A)Immunofluorescence showed the localization and expression of the senescence marker protein p16 in the retina of Control mice and DM mice; quantitative analysis of the proportion of p16 expression‐positive cells in RPE cells, *n* = 4. B)The expression of p16 and p21 was analyzed by Western blot on ARPE‐19 cells cultured in gradients of different glucose concentrations for 48 h, *n* = 3. C) Representative images of SA‐β‐gal staining of ARPE‐19 cells cultured in gradients of different glucose concentrations for 48 h, *n* = 3. D) CCK8 detection and evaluation of the effects of stimulation with different glucose concentrations for 48 h on ARPE‐19 cell proliferation, *n* = 6. *Statistically different from normal glucose concentration (5.5mm). E) RNA‐seq analysis of ARPE‐19 cells under control (5.5mm glucose + 32mm mannitol, *n* = 5) and high glucose (37.5mm, *n* = 5) conditions, combined with primate DR model transcriptome (GSE160617), showing consistent PDZK1 downregulation (|FC| > 1.5, *p* < 0.05). (F) Western blot analysis verified PDZK1 expression in control group and high glucose (HG) group in ARPE‐19 cells, *n* = 4. All results were analyzed by a two‐tailed unpaired *t* test or one‐way analysis of variance (ANOVA) followed by Tukey's multiple comparison test. Data are expressed as mean ± standard deviation (SD). ^*^ *P* < 0.05, ^**^ *P* < 0.01, ^***^ *P* < 0.001, ^****^ *P* < 0.0001.

In order to study whether DM can induce senescence in RPE cells, we established a STZ‐induced diabetic mouse model (DM group) and found that compared with the control group, the senescence marker p16 in the DM group was significantly upregulated in RPE (**Figure** **1**A), which was consistent with the performance of RPE in DR patients.^[^ ^4^ ^]^ To further confirm high‐glucose‐induced senescence in RPE cells, we examined the expression of established senescence markers p16, p21, and SA‐β‐gal activity, which showed concentration‐dependent increases (Figure 1B,C). Consistent with the expression trends of these senescence markers, the proliferation activity of RPE cells significantly decreased when stimulated by glucose concentrations of 37.5mM and higher (Figure 1D). Therefore, we selected RPE cells stimulated with 37.5mM glucose for 48h as an in vitro model to simulate diabetes for transcriptome sequencing and subsequent research.

Based on our sequencing results, combined with the RPE cell transcriptome (GSE160617) data of the primate early DR model (spontaneous type 2 diabetes combined with retinal functional changes), joint analysis screened out differentially expressed genes and found that PDZK1 expression was significantly reduced (Figure 1E). This result was verified in the RPE cell model (Figure 1F).

### 2.2 PDZK1 Inhibited High‐Glucose‐Induced RPE Cell Senescence and Transport Dysfunction

**Figure 2:**
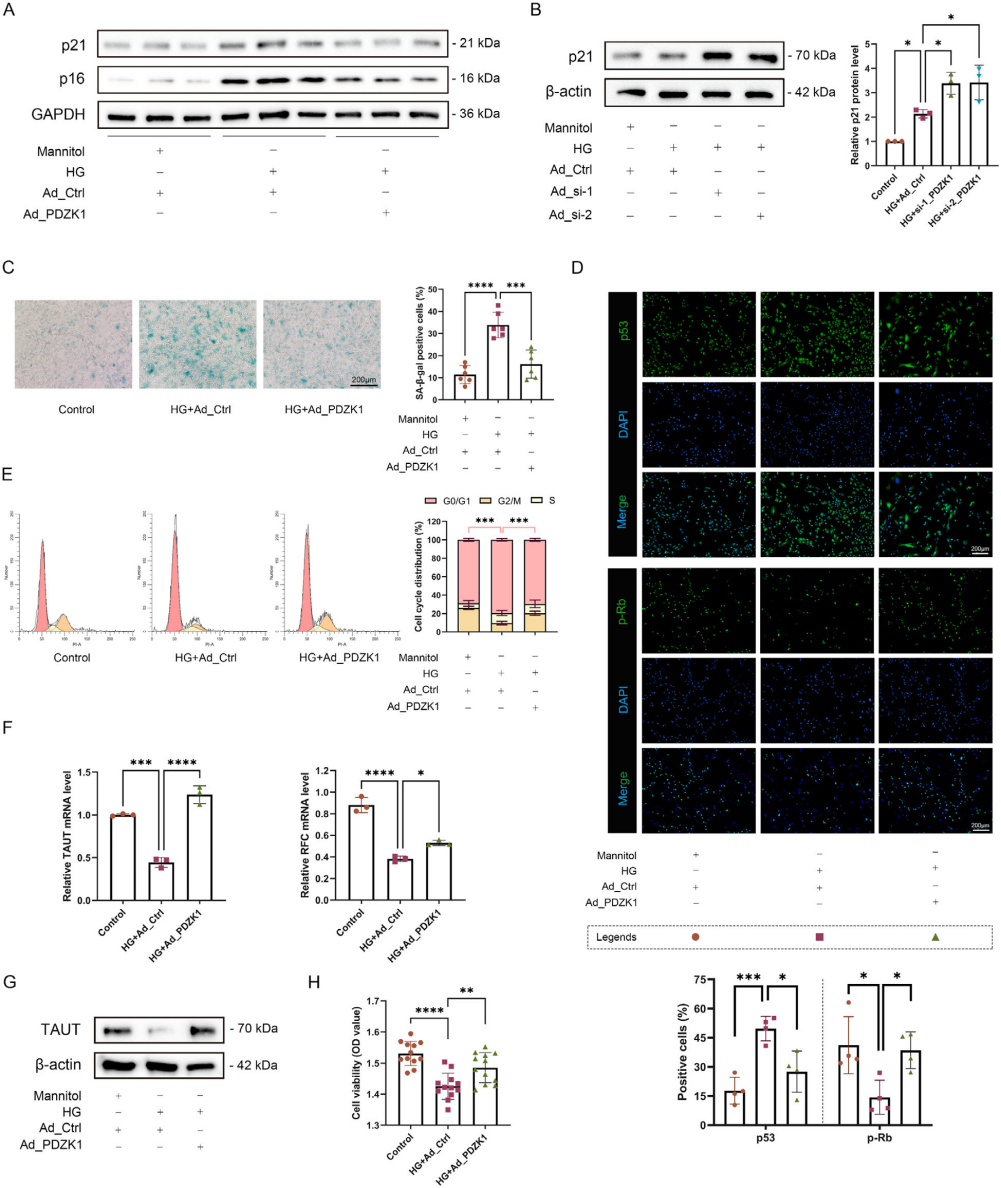
PDZK1 inhibited high‐glucose‐induced RPE cell senescence and transport dysfunction. The experimental groups included the control group (5.5mm glucose + 32mm mannitol + Ad_Ctrl), HG group (37.5mm glucose + Ad_Ctrl), PDZK1 overexpression group (37.5mm glucose + Ad_PDZK1) and PDZK1 knockdown group (37.5mm glucose + Ad_si‐1/2). A) Overexpression of PDZK1. Western blot analysis of p16 and p21 expression levels in ARPE‐19 cells, *n* = 3. B) Knockdown of PDZK1. Western blot analysis of p21 expression levels in ARPE‐19 cells, *n* = 3. C) Representative images of SA‐β‐gal staining in ARPE‐19 cells, *n* = 6. D) Immunofluorescence analysis of p53 and p‐Rb expression in ARPE‐19 cells from different experimental groups, *n* = 4. E) Flow cytometry analysis of ARPE‐19 cell cycle distribution and G0/G1 ratio, *n* = 3. F) qRT‐PCR analysis of TAUT and RFC mRNA expression levels in ARPE‐19 cells, *n* = 3. G) Western blot analysis of TAUT protein expression levels in ARPE‐19 cells, *n* = 3. H) CCK8 detection of ARPE‐19 cell viability, *n* = 12. All results were analyzed by one‐way ANOVA followed by Tukey's multiple comparison test. Data are expressed as mean ± SD. ^*^ *P* < 0.05, ^**^ *P* < 0.01, ^***^ *P* < 0.001, ^****^ *P* < 0.0001.

To clarify the role of PDZK1 in the senescence of RPE cells induced by high glucose, we performed both gain and loss‐of‐function studies. Overexpression of PDZK1 reversed high‐glucose‐evoked increases in p16, p21, p53, and SA‐β‐gal activity, together with the accompanying decrease in p‐Rb, in ARPE‐19 cells (**Figure** **2**A,C,D) as well as in human primary RPE cells (Figure S1A,B, Supporting Information). While Knockdown of PDZK1 further exacerbated the high‐glucose‐induced increase in p21 expression in RPE cells (Figure 2B). Similarly, overexpression of PDZK1 rescued RPE cell cycle arrest and decreased proliferation activity caused by high glucose (Figure 2H).

In addition, since cell senescence is usually accompanied by dysfunction, we found that the key transporters of RPE, taurine transporter (TAUT) and reduced folate carrier (RFC), were down‐regulated under high glucose stimulation, while overexpression of PDZK1 reversed this trend (Figure 2F,G). These results indicate that PDZK1 can alleviate high‐glucose‐induced senescence and a decrease in the transport function of RPE cells.

### 2.3 PDZK1 may Inhibit RPE Cell Senescence by Improving High‐Glucose‐Induced Oxidative Stress and Autophagy Dysfunction

**Figure 3:**
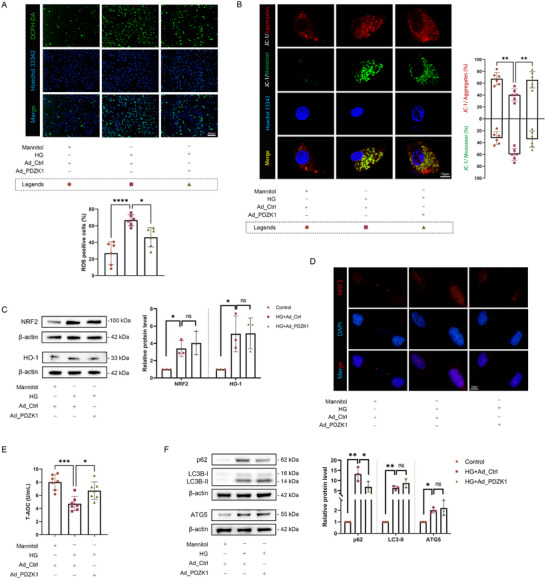
PDZK1 may inhibit RPE cell senescence by improving high‐glucose‐induced oxidative stress and autophagy dysfunction. A) ROS fluorescent probe DCFH‐DA was used to detect ROS levels in ARPE‐19 cells, *n*=6. B) Measurement of mitochondrial membrane potential changes in ARPE‐19 cells using JC‐1 fluorescent probe, *n* = 6. C) Western blot analysis of the expression of NRF2 and HO‐1 in ARPE‐19 cells, *n* = 3. D) Representative images of immunofluorescence assessment of NRF2 nuclear translocation in ARPE‐19 cells, n = 3. E) Total antioxidant capacity (T‐AOC) detection results of ARPE‐19 cells in different experimental groups, *n* = 7. F) Western blot analysis of p62, LC3B, and ATG5 expression in ARPE‐19 cells, *n* = 3. All results were analyzed by one‐way ANOVA followed by Tukey's multiple comparison test. Data are expressed as mean ± SD. ^*^ *P* < 0.05, ^**^ *P* < 0.01, ^***^ *P* < 0.001, ^****^ *P* < 0.0001.

SIPS are induced by various stressors.^[^ ^6^ ^]^ Oxidative stress and autophagy are not only closely related to cellular senescence, but are also one of the main pathogenic factors involved in the pathogenesis of DR.^[^ ^25^ ^]^ Therefore, we evaluated the reactive oxygen species (ROS) levels and changes in mitochondrial membrane potential in RPE cells under high glucose conditions, and found that overexpression of PDZK1 could alleviate the increase in ROS and depolarization of mitochondrial membrane potential caused by high glucose (**Figures** **3**A,B; S1C, Supporting Information). Interestingly, NRF2/HO‐1, as an important antioxidant stress pathway, was significantly activated under high glucose conditions (upregulation of NRF2, HO‐1 expression and increased NRF2 nuclear translocation), however, the total antioxidant capacity (T‐AOC) decreased; the overexpression group restored its T‐AOC while activating the NRF2/HO‐1 pathway (Figure 3C–E). The overall results show that high glucose stimulation can cause increased oxidative stress levels and reactive activation of the NRF2/HO‐1 antioxidant pathway in RPE cells, but the T‐AOC decreases. This trend can be reversed by the overexpression of PDZK1.

Autophagy can maintain ROS levels at tolerable levels by clearing damaged mitochondria.^[^ ^25^ ^]^ The results of this experiment showed that the LC3B‐II/I ratio of RPE cells in the high glucose group was increased, and ATG5 was upregulated. However, p62 protein accumulated, which means that autophagy flux was impaired. At the same time, the PDZK1 overexpression group successfully increased autophagy flux to cope with high‐glucose‐induced oxidative stress (Figure 3F).

### 2.4 PDZK1 Alleviated High‐Glucose‐Induced RPE Cell Senescence by Inhibiting the mTOR Pathway

**Figure 4:**
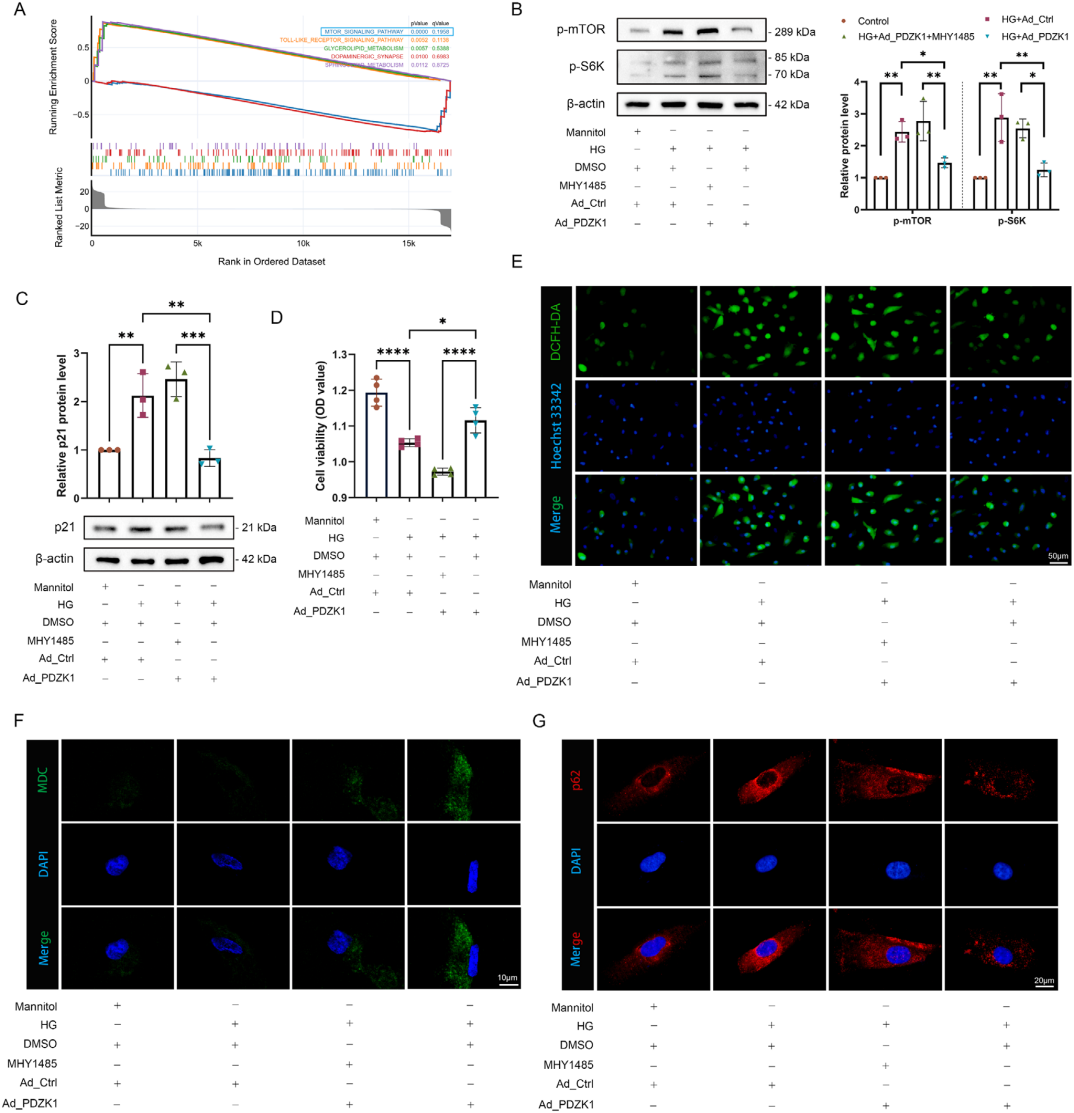
PDZK1 alleviated high‐glucose‐induced RPE cell senescence by inhibiting the mTOR pathway. A) GSEA analysis shows that the mTOR pathway changes significantly in PDZK1‐overexpressing ARPE‐19 cells compared with the HG group. B) Western blot analysis of p‐mTOR and p‐S6K expression levels in ARPE‐19 cells, *n* = 3. C) Western blot analysis of p21 expression levels in ARPE‐19 cells, *n* = 3. D) CCK8 detection of ARPE‐19 cell viability, *n* = 4. E) Representative images of ROS levels detected in ARPE‐19 cells using the ROS fluorescent probe DCFH‐DA, *n* = 3. F) Representative images of autophagy assessed using MDC fluorescent staining, *n* = 3. G) Representative images of immunofluorescence detection of p62 expression in ARPE‐19 cells, *n* = 3. All results were analyzed by one‐way ANOVA followed by Tukey's multiple comparison test. Data are expressed as mean ± SD. ^*^ *P* < 0.05, ^**^ *P* < 0.01, ^***^ *P* < 0.001, ^****^ *P* < 0.0001.

In order to explore the protective mechanism of PDZK1 on RPE cells under high‐glucose conditions, we performed Gene set enrichment analysis (GSEA) analysis on the high‐glucose group and the overexpression PDZK1 group, and the results showed that the mTOR pathway changed most significantly (**Figure** **4**A). The experimental results further confirmed that high glucose induced the up‐regulation of p‐mTOR and p‐S6K expression in RPE cells, and overexpression of PDZK1 alleviated this change; administration of the specific mTOR activator MHY1485 abolished the alleviation of the senescence phenotype mediated by overexpression of PDZK1(Figure 4B–D). Consistently, the decrease in ROS and increase in autophagic flux mediated by overexpression of PDZK1 were abolished after activation of the mTOR pathway by MHY1485 (Figure 4E–G). In general, these results indicate that PDZK1 alleviates high‐glucose‐induced cell senescence, oxidative stress, and impaired autophagy flux by inhibiting mTOR pathway activity.

### 2.5 PDZK1 Interacts with 14‐3‐3ε to Regulate the mTOR Pathway

**Figure 5:**
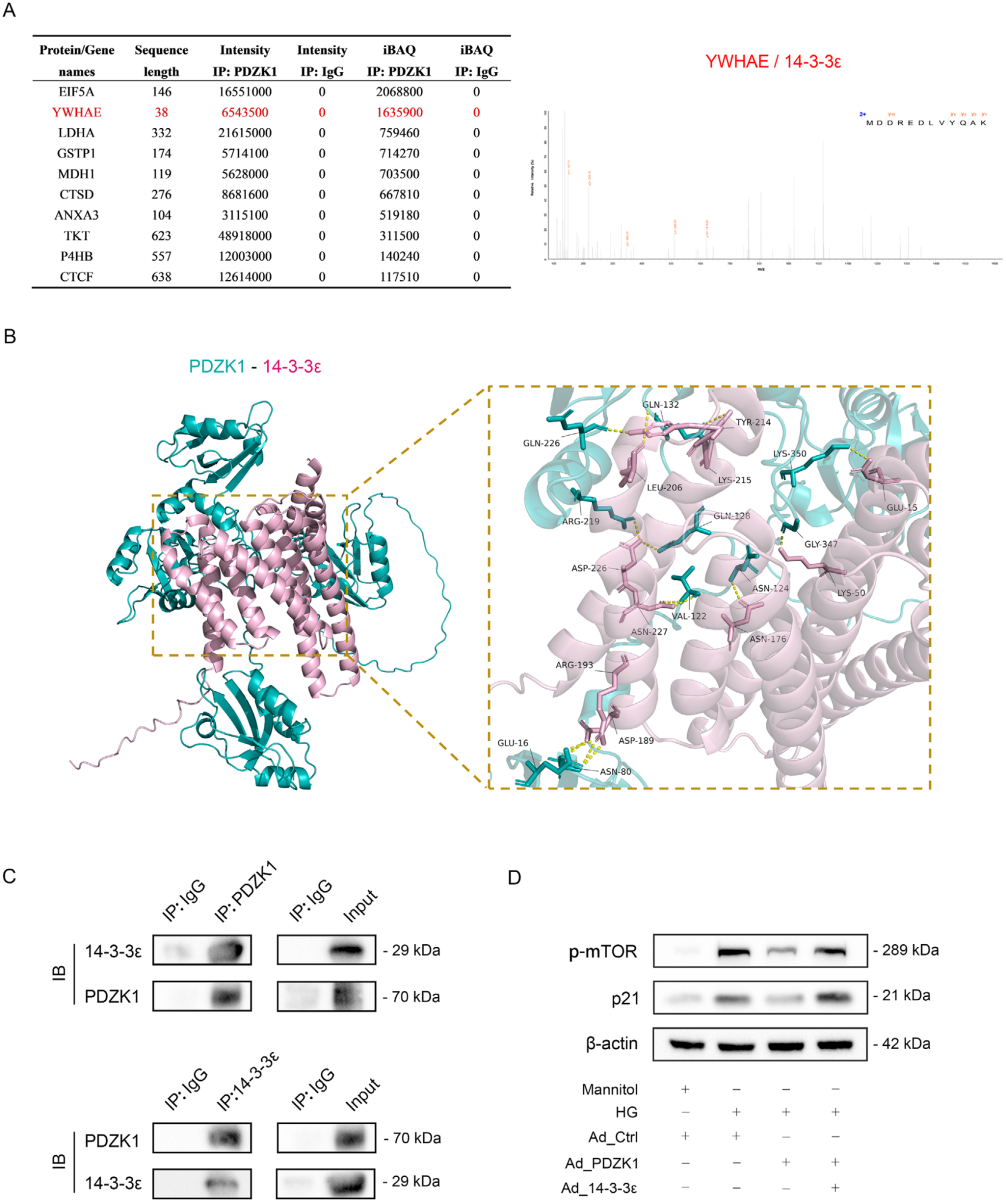
PDZK1 interacts with 14‐3‐3ε to regulate the mTOR pathway. A) Co‐immunoprecipitation (Co‐IP) of PDZK1 from ARPE‐19 cell lysates followed by liquid chromatography‐tandem mass spectrometry (LC‐MS/MS) identified 14‐3‐3ε (YWHAE) as a PDZK1‐binding partner. B) Structural prediction of PDZK1 (UniProtKB ID: Q5T2W1)‐14‐3‐3ε (UniProtKB ID: P62258) interaction using AlphaFold 3 (visualized with PyMOL). C) Co‐IP validation of PDZK1‐14‐3‐3ε interaction in ARPE‐19 cells, *n* = 3. D) Western blot analysis demonstrating the effects of PDZK1‐14‐3‐3ε interaction on mTOR pathway activation (p‐mTOR) and senescence phenotype (p21) in ARPE‐19 cells, *n* = 3.

To further investigate the molecular mechanism by which PDZK1 regulates the mTOR pathway in RPE cells, we performed co‐immunoprecipitation coupled with mass spectrometry (Co‐IP/MS) analysis and identified 14‐3‐3ε (YWHAE) as a novel PDZK1‐interacting protein (**Figure** **5**A). To characterize this interaction, we conducted computational modeling of the predicted PDZK1–14‐3‐3ε interface using AlphaFold 3, which revealed key amino acids involved in protein‐protein interactions (Figure 5B).^[^ ^26^ ^]^ Subsequent co‐immunoprecipitation (co‐IP) assays confirmed the physical interaction between PDZK1 and 14‐3‐3ε in RPE cells (Figure 5C). Immunofluorescence revealed colocalization of PDZK1 and 14‐3‐3ε in human primary RPE cells (Figure 1E). The 14‐3‐3ε protein typically acts as a positive regulator of the mTOR pathway.^[^ ^27^, ^28^ ^]^ We further observed that overexpression of 14‐3‐3ε in RPE cells counteracted the inhibitory effects of PDZK1 on the mTOR pathway and the senescence phenotype (Figure 5D; Figure S1D, Supporting Information). These results indicate that PDZK1 protects against RPE cell senescence by targeting the 14‐3‐3ε‐mTOR pathway.

### 2.6 Overexpression of PDZK1 Inhibited RPE Cell Senescence and Alleviated Early DR Lesions in Diabetic Mice

**Figure 6:**
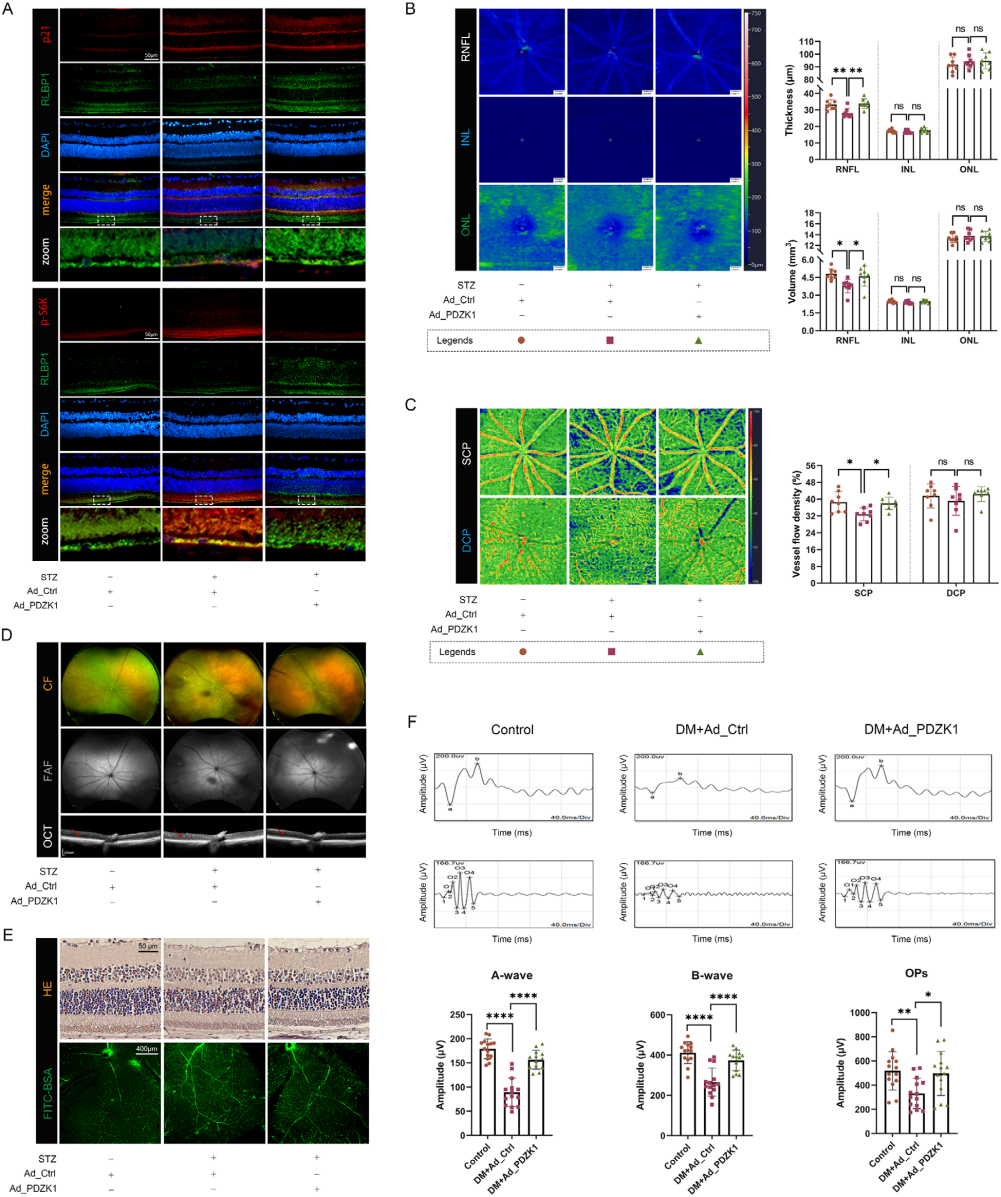
Overexpression of PDZK1 inhibited RPE cell senescence and alleviated early DR lesions in diabetic mice. (A) Immunofluorescence showed representative images of the localization and expression of RPE cell markers RLBP1 and p21, p‐S6K in the retina of mice in each group, *n* = 5. B) OCTA detected the average thickness and total volume of RNFL, INL, and outer nuclear layer (ONL) in the retina of mice in each group, *n* = 8. C) OCTA analysis of blood flow density of SCP and deep capillary plexus layer (DCP) in mouse retina, *n* = 8. D) Representative wide‐angle color fundus images (CF), fundus autofluorescence images (FAF), and OCT scan images of mice in each group (red arrows indicate density comparison of ONL in each group), *n* = 5. E) Representative images of mouse retinal sections after oxidative decolorization, HE staining, and intravenous injection of FITC‐BSA to detect retinal vascular leakage, *n* = 3. F) The amplitudes of a‐wave, b‐wave, and Ops of mice in each group were analyzed by visual electrophysiological examination (dark adaptation 3.0), *n* = 14. All results were analyzed by one‐way ANOVA followed by Tukey's multiple comparison test. Data are expressed as mean ± SD. ^*^ *P* < 0.05, ^**^ *P* < 0.01, ^***^ *P* < 0.001, ^****^ *P* < 0.0001.

Based on the protective effect of PDZK1 in the high‐glucose‐induced RPE cell model, we established STZ‐induced diabetic mice as an early DR animal model for further verification. Consistent with the results of in vitro experiments, the RPE of the STZ group showed up‐regulation of p21 expression and activation of the mTOR pathway (up‐regulation of p‐S6K expression), and overexpressing PDZK1 alleviated these changes (**Figure** **6**A). Compared with the control group, the color fundus photos of mice in the STZ group showed blurred fundus blood vessels and more obvious punctate hyperfluorescent lesions in fundus autofluorescence, reflecting an increase in abnormal RPE cells. However, the above changes in the overexpression group were alleviated (Figure 6D). Further examination by optical coherence tomography angiography (OCTA) found that the the volume and average thickness of the retinal nerve fiber layer (RNFL) in diabetic mice were reduced, and the blood flow density of superficial capillary plexus layer (SCP) was also significantly reduced. Overexpression of PDZK1 also showed a protective effect gratifyingly (Figure 6B,C). The vascular leakage experiment showed that compared with the control group, no obvious fluorescence leakage was observed in the retina of the STZ group, but the vascular morphology was more tortuous and blurred, while the vascular morphology of the overexpression group was closer to the control group (Figure 6E). Similarly, Hematoxylin and eosin (HE) staining showed that the RNFL, inner nuclear layer (INL), and RPE layers of diabetic mice were disorganized or even missing compared with the control group, while the overexpression group alleviated the above lesions (Figure 6E).

Retinal electrophysiological changes are one of the early changes in DR. Here, we demonstrated that overexpression of PDZK1 can alleviate the decrease in a wave, b wave, and oscillatory potential wave volts in diabetic mice during the dark adaptation 3.0 examination (Figure 6F). Overall, these data indicate that overexpression of PDZK1 can inhibit RPE cell senescence and alleviate early DR lesions in structure and function in diabetic mice.

### 2.7 Nutlin‐3a Eliminated Senescent RPE Cells and Alleviated Early DR Lesions in Diabetic Mice

**Figure 7:**
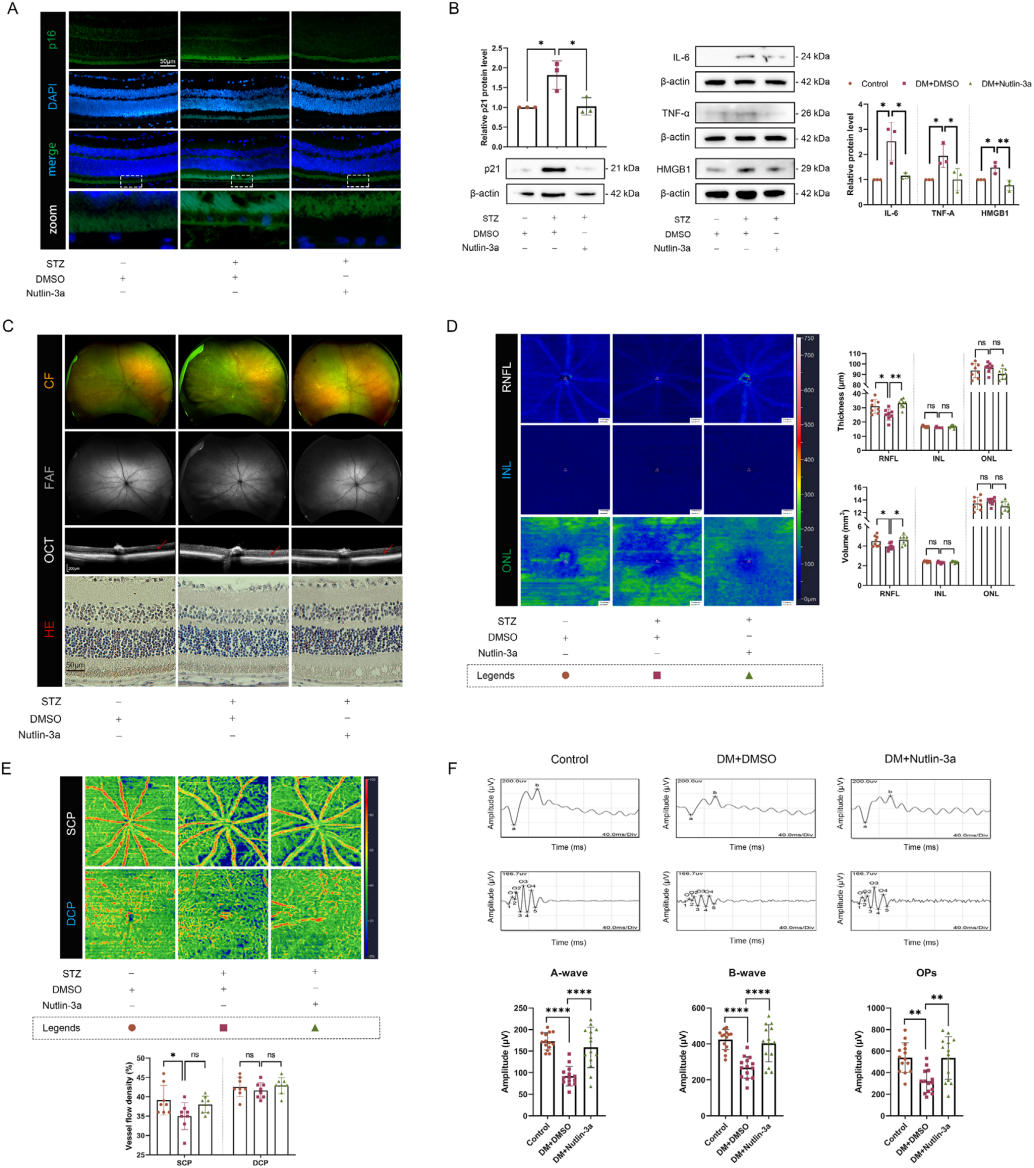
Nutlin‐3a eliminated senescent RPE cells and alleviated early DR lesions in diabetic mice. A) Representative images of immunofluorescence showing the localization and expression of p16 in the retina of mice in each group, *n* = 4. B) Western blot analysis of p21 and SASP (IL‐6, TNF‐α, and HMGB1) expression levels in mouse retinal tissue (including RPE/choroid complex), *n* = 3. C) Representative wide‐angle color fundus images (CF), fundus autofluorescence images (FAF), OCT scan images (red arrows indicate density comparison of ONL in each group), and retinal HE staining images of mice in each group, *n* = 4. (D) OCTA detected the average thickness and total volume of RNFL, INL, and ONL in the retina of mice in each group, *n* = 8. (E) OCTA analysis of blood flow density of SCP and DCP in mouse retina, *n* = 8. F) The amplitudes of a‐wave, b‐wave, and Ops of mice in each group were analyzed by visual electrophysiological examination (dark adaptation 3.0), *n* = 14. All results were analyzed by one‐way ANOVA followed by Tukey's multiple comparison test. Data are expressed as mean ± SD. ^*^ *P* < 0.05, ^**^ *P* < 0.01, ^***^ *P* < 0.001, ^****^ *P* < 0.0001.

Nutlin‐3a is confirmed to be a senolytic that can target and eliminate senescent RPE cells.^[^ ^24^ ^]^ To explore the exact role of RPE cell senescence in DR, we administered intravitreal injection of Nutlin‐3a to STZ‐induced diabetic mice to eliminate senescent RPE cells (**Figure** **7**A) and observed its possible beneficial effects. Compared with the STZ group, the expression of p16 in the RPE of the Nutlin‐3a group was down‐regulated, and the levels of p21 and SASP (IL‐6, TNF‐α and HMGB1) in the retinal tissue were also reduced (Figure 7B). At the same time, it was surprisingly found that the fundus vascular morphology and punctate hyperfluorescent lesions were improved in diabetic mice treated with Nutlin‐3a, which means that Nutlin‐3a alleviated diabetes‐induced retinopathy (Figure 7C). HE staining of mouse retinas showed that Nutlin‐3a treatment could alleviate changes such as disordered arrangement and even deletion of RPE cells in diabetic mice (Figure 7C). OTCA examination showed that Nutlin‐3a alleviated the diabetes‐induced decrease in the thickness and volume of the RNFL layer (Figure 7D,E).

In terms of retinal function, Nutlin‐3a can alleviate the decrease in a‐wave, b‐wave, and oscillatory potential wave volts in diabetic mice during the dark adaptation 3.0 examination (Figure 7F). Overall, these data indicate that Nutlin‐3a alleviates early DR pathology in diabetic mice by clearing senescent RPE cells.

## 3 Discussion

Retinal microcirculation disorder is an early characteristic of DR and an important link in the occurrence and development of DR. However, in contrast to the retinal circulation, the choroidal circulation supplies ≈85% of the retina's blood flow. And the RPE, as the key hub between the choroid and neural retina, plays a vital role in this process. In fact, RPE dysfunction not only precedes retinal vascular changes in the diabetic retina but also occurs before DR changes.^[^ ^29^ ^]^ Clinical electrooculogram study has shown that the RPE is very sensitive to changes in blood glucose.^[^ ^30^ ^]^ In vitro study has revealed that high glucose can induce ARPE‐19 cells' senescence in the short term (48 h), and cell senescence occurred before apoptosis (the result of long‐term high glucose damage).^[^ ^31^ ^]^ These results were similar to our observations in the high‐glucose‐induced RPE cell model and STZ‐induced diabetic mice. A new and interesting question is then what is the specific impact of RPE cell senescence on DR and the molecular mechanism?

Transcriptomic analysis suggested that PDZK1 is a potential key gene that drives the occurrence and development of DR in the early stages. Our results showed that knockdown of PDZK1 exacerbated high‐glucose‐induced senescence in RPE cells. In turn, overexpression of PDZK1 could significantly downregulate the expression of multiple senescence marker proteins and improve cell proliferation activity. Importantly, as a multifunctional membrane scaffolding protein, PDZK1 organizes transporters and regulates a variety of transporter functions, playing a crucial role in cell proliferation, differentiation, and ion transport. Taurine is the most abundant amino acid in the retina and is an important antioxidant molecule along with folic acid.^[^ ^32^, ^33^ ^]^ Studies have pointed out that taurine deficiency is a driving factor of aging, and taurine intake may alleviate the visual function decline of diabetic rats by activating TAUT.^[^ ^34^, ^35^ ^]^ Another study found that the expression and activity of RFC were significantly decreased in RPE cells of STZ‐induced diabetic mice.^[^ ^36^ ^]^ We found that TAUT and RFC in RPE cells were significantly down‐regulated under high‐glucose stimulation, and overexpression of PDZK1 alleviated this trend. Taken together, our results indicate that PDZK1 alleviates high‐glucose‐induced senescence and a decrease in transport function in RPE cells.

The free radical theory of aging emphasizes the role of ROS‐induced cell damage in promoting aging. Under hyperglycemic conditions, excessive accumulation of ROS induces mitochondrial damage, lipid peroxidation, and inflammation, further resulting in structural and functional alterations in the retina.^[^ ^37^ ^]^ Correspondingly, autophagy is an important biological process to respond to various stresses and maintain cellular homeostasis, and is also a potential target for anti‐aging treatment.^[^ ^38^, ^39^ ^]^ Studies have shown that autophagy maintains ROS levels at tolerable levels by removing partially depolarized mitochondria, and dysregulated autophagy may play a key role in the onset and progression of DR.^[^ ^25^ ^]^ In this study, we observed that under high glucose conditions, ROS levels and the proportion of depolarized mitochondria increased in RPE cells, accompanied by autophagy activation but impaired autophagy flux. These may be the more direct reasons for the senescence process of RPE cells induced by high glucose. After overexpressing PDZK1, the autophagy flux of RPE cells increased smoothly under high glucose conditions, and the level of oxidative stress also decreased. However, the mechanism by which PDZK1 reduces ROS remains incompletely understood. Our data suggest that PDZK1 may sustain cellular antioxidant capacity independently of the NRF2/HO‐1 pathway. Notably, previous studies have proposed that PDZK1 regulates OCTN transporters responsible for renal uptake of the antioxidants ergothioneine and L‐carnitine, thereby reducing renal ROS production.^[^ ^40^ ^]^ This suggests a potential parallel mechanism in RPE cells, where PDZK1 might enhance antioxidant defenses through transporter‐mediated pathways (possibly involving OCTN, TAUT, or RFC transporters).

GSEA analysis suggested that the above‐mentioned protective effects of PDZK1 are mediated through inhibition of the mTOR pathway, a conclusion further supported by experimental results in both RPE cells and diabetic mouse models. In the field of ophthalmology research, mTOR pathway activation plays an important role in high‐glucose‐mediated RPE cell damage.^[^ ^41^, ^42^ ^]^ Many evidences show that during the pathogenesis of DR, the activated mTOR pathway can inhibit autophagy, regulate apoptosis, and promote the expression of inflammatory factors.^[^ ^21^ ^]^ Recent research has indicated that inhibiting the mTOR and downstream S6K signaling pathways holds promise as a strategy for anti‐aging treatments.^[^ ^43^ ^]^

Further mechanistic investigations confirmed that PDZK1 physically interacts with 14‐3‐3ε, a key regulatory protein. The 14‐3‐3 protein family consists of highly conserved acidic proteins that play crucial roles in cellular signaling by binding and modulating the activity of their client proteins. Notably, dysregulation of 14‐3‐3 target proteins has been implicated in various human diseases, including DM and cancer.^[^ ^23^ ^]^ In our study, overexpression of 14‐3‐3ε in RPE cells effectively rescued PDZK1‐mediated suppression of mTOR signaling and cell senescence, suggesting that the PDZK1‐14‐3‐3ε‐mTOR axis plays a critical role in high‐glucose‐induced RPE cell senescence.

We adopted two strategies to further elucidate the relationship between RPE cell senescence and DR. On the one hand, we inhibited RPE senescence in diabetic mice by intravitreal injection of overexpressed PDZK1 lentivirus and observed early DR changes. Eliminating sources of SASP by removing senescent cells from tissues has been reported to improve tissue homeostasis and ameliorate multiple diseases.^[^ ^24^ ^]^ So on the other hand, we chose intravitreal injection of Nutlin‐3a to eliminate senescent RPE cells in diabetic mice. It was gratifying that both strategies have achieved positive therapeutic effects, including the down‐regulation of retinal senescence marker proteins, improvement of RNFL thickness and volume, etc. In particular, the improvement of retinal electrophysiology provided strong evidence that RPE cell senescence is involved in the occurrence of DR, and reveals the important role of PDZK1 in this process. Since the preliminary study adopted intravitreal injection, the protective effect observed above may not only be mediated by RPE cells, but may also involve the synergistic effect of other cell types. Further evidence is needed in the future to reveal the specific roles of PDZK1 and RPE cell senescence in DR.

**Figure 8:**
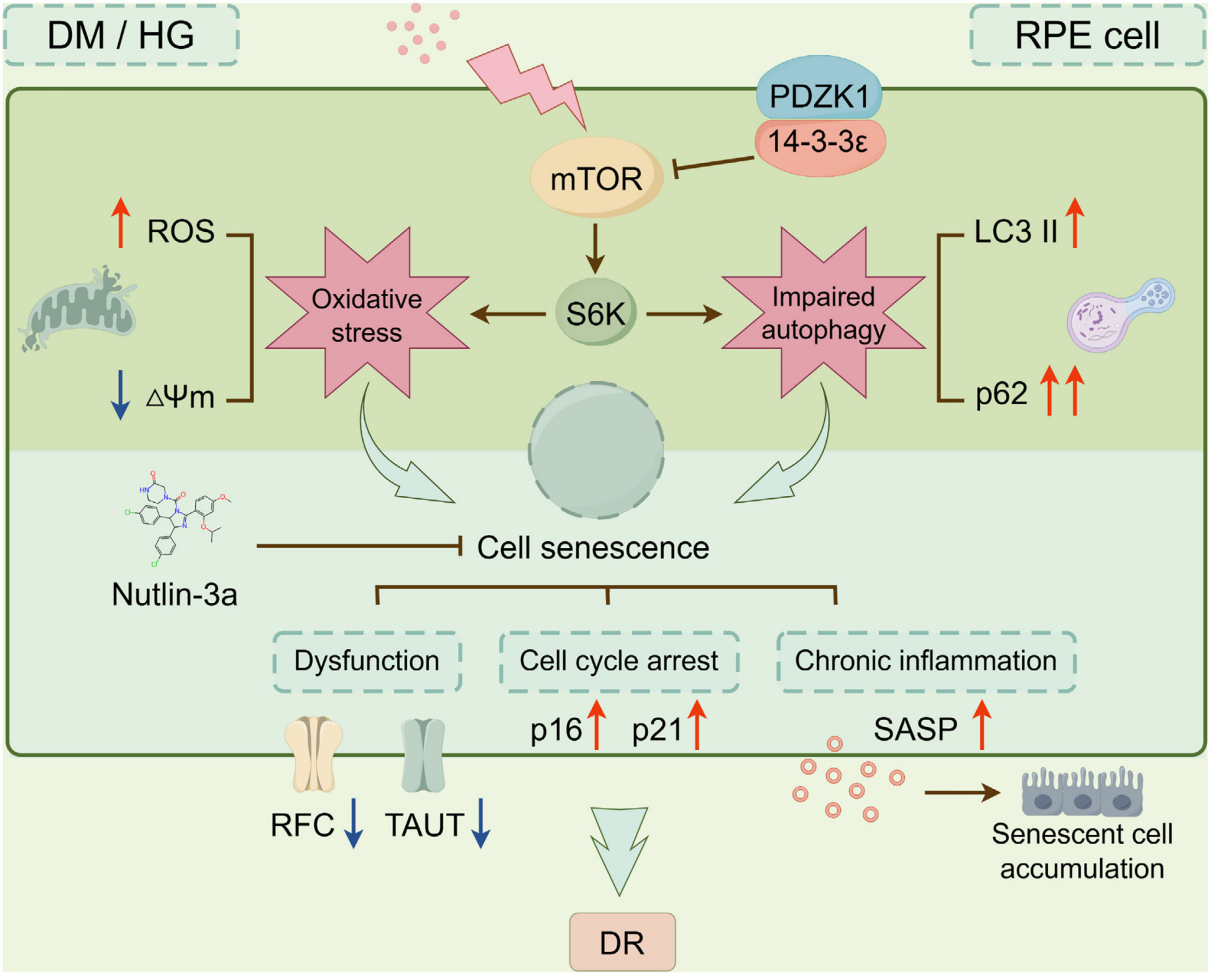
Schematic diagram: PDZK1 inhibits oxidative stress, impaired autophagy flux, and cell senescence by suppressing 14‐3‐3ε‐mTOR pathway in RPE cells, and Nutlin‐3a eliminates senescent RPE cells to mitigate early DR lesions.

**Figure** **8** presents a schematic summary of the key findings from this study. Our results demonstrate that PDZK1 ameliorates high‐glucose‐induced RPE cell senescence through modulation of the 14‐3‐3ε‐mTOR pathway, which is associated with attenuating oxidative stress and restoring autophagy flux. Importantly, we provide experimental evidence that targeting RPE senescence—specifically, PDZK1 overexpression to prevent RPE senescence and Nutlin‐3a administration to clear senescent cells—can effectively mitigate early DR pathology in diabetic mouse models. To our knowledge, this study represents the first direct demonstration that RPE cell senescence acts as a pathogenic contributor to the onset and progression of DR, thereby identifying novel therapeutic targets and strategies for DR intervention.

## 4 Experimental Section

### Cell Culture and Transfection

The ARPE‐19 cell line (RRID: CVCL_0145) was obtained from the Cell Bank of the Chinese Academy of Sciences, and human primary RPE cells (HUM‐iCELL‐m011) were purchased from iCell Bioscience (Shanghai, China). Authentication was performed by short tandem repeat (STR) profiling, and all cells were verified mycoplasma‐free by the supplier. Cells were maintained in DMEM supplemented with 10% fetal bovine serum and 1% penicillin–streptomycin at 37 °C in a humidified 5% CO_2_ incubator. If there were no special instructions, cells were cultured at a 5.5 mm d‐glucose and 32 mM mannitol concentration for control, while 37.5 mM d‐glucose for high glucose (HG). The above‐mentioned culture conditions and centrifugation settings were applied throughout the study unless mentioned otherwise. Specifically, MHY1485 (MedChemExpress) was used at a working concentration of 5 µm.

The human PDZK1 overexpression lentivirus and control lentivirus were purchased from Tsingke Biotech. Cells were cultured to 50% confluence and transduced with lentivirus (MOI = 10) following the manufacturer's instructions. After 48 h of transduction, 4µg/ml‐1 puromycin was added to the culture medium for stable cell line selection.

The human PDZK1 and YWHAE overexpression plasmids were purchased from MiaoLing Plasmid Platform, and the human PDZK1 specific siRNA was obtained from KeyGEN BioTECH. Transfections were performed using the KeygenMax 3000 Transfection Reagent (KeyGEN BioTECH) according to the manufacturer's instructions. The siRNA sequences were as follows (5’→3’):

PDZK1 siRNA‐1: GGAGCAAGGUUUGAGUGAUTT;

PDZK1 siRNA‐2: GUCAAAUCAUCAAGGACAUTT.

### Animals

Eight‐week‐old male C57BL/6J mice were purchased from Guangzhou Ruige Biological Technology Co.,Ltd. The Southern Medical University Committee Animal Care and Use Committee approved all protocols in this study. Diabetes was induced by intraperitoneal injection with 55 mg/kg streptozotocin (Sigma) dissolved in sodium citrate buffer (pH = 4.5) for 5 consecutive days, whereas control mice were treated with sodium citrate buffer only. One week later, the blood glucose level was determined, and readings ≥16.7 mM were considered to indicate diabetes.

The mouse PDZK1 overexpression lentivirus and control lentivirus were obtained from KeyGEN BioTECH. One week post‐diabetes induction, mice were anesthetized by intraperitoneal injection of 2% tribromoethanol (300 mg kg^−1^) and then administered an intravitreal injection of 1 µL lentivirus every month or 20 ng µL^−1^ Nutlin‐3a (Macklin) every week using the 33‐gauge Hamilton microsyringe. The Mice were sacrificed 16 weeks after the first intraperitoneal injection.

### Antibodies

Anti‐GAPDH (P30008M, Abmart), anti‐β‐actin (EM21002, HUABIO), anti‐p16 (10883‐1‐AP, ProteinTech), anti‐p21 (HA500005, HUABIO), anti‐p53 (ET1602‐38, HUABIO), anti‐p‐Rb (ET1602‐36, HUABIO), anti‐NRF2 (HA721432, HUABIO), anti‐HO‐1 (HA721854, HUABIO), anti‐p62 (T55546F, Abmart), anti‐LC3B (T55992F, Abmart), anti‐ATG5 (T55766F, Abmart), anti‐p‐mTOR (T56571F, Abmart), anti‐p‐S6K (HA721803, HUABIO), anti‐14‐3‐3ε (JC821430S, Abmart), anti‐RLBP1 (MG343186S, Abmart), anti‐IL‐6 (TD6087, Abmart), anti‐TNF‐α (WL01581, Wanleibio), anti‐HMGB1 (10829‐1‐AP, ProteinTech), anti‐PDZK1 (10507‐2‐AP, ProteinTech), HRP Conjugated Goat anti‐Rabbit IgG (HA1001, HUABIO), HRP‐conjugated Goat Anti‐Mouse IgG (SA00001‐1, ProteinTech), iFluor™ 488 Conjugated Goat anti‐rabbit IgG (HA1121, HUABIO), Multi‐rAb CoraLite® Plus 555‐Goat Anti‐Mouse Recombinant Secondary Antibody (RGAM003, ProteinTech).

### Western Blot Analysis

Cell or tissue samples were lysed in RIPA buffer containing protease inhibitors and phosphatase inhibitors. Protein concentrations were measured with the BCA protein assay kit (ThermoFisher). Proteins were separated on 4–20% precast PAGE Gel (KeyGEN BioTECH) and transferred to PVDF membranes (Millipore). The membranes were blocked with 5% nonfat milk, followed by overnight incubation with primary antibodies at 4 °C. After washing, Secondary antibodies were incubated for 1 h at room temperature. The protein bands were visualized with the ECL Super Signal (GBCBIO) and the Tanon Imaging System. The gray scale analysis of immunoblots was quantified with Image J software. And the relative levels of target proteins were normalized by GAPDH or β‐actin. The Co‐Immunoprecipitation Kit was purchased from Abmart (Shanghai, China) and used according to the manufacturer's protocol.

### CCK‐8 Assay

According to the manufacturer's protocol, cells were seeded at a density of 2 × 10^3^ into 96‐well plates and incubated for 24h. Then, a stimulator with specific drugs was administered to the cells, and incubation was continued for another 48h. Fresh medium containing 10% CCK‐8 solution (GBCBIO) was replaced and incubated for 1–2 h at 3 °C and then read at 450 nm with a microplate reader.

### SA‐β‐Gal Assay

The SA‐β‐gal positive cells were measured using a senescence β‐gal staining kit (Beyotime). In brief, cells were washed with PBS and subsequently fixed for 15 min at room temperature using the fixation solution provided in the kit. The cells were rinsed again with PBS and then incubated overnight at 37 °C with the working solution. Images were acquired under an inverted microscope (Olympus).

### Cell‐Cycle Analysis

Cells were collected and fixed in 70% precooled ethanol overnight at 4 °C. After washing off the fixing solution with PBS, the cells were incubated with PI/RNaseA staining working solution from the Cell Cycle Detection Kit (KeyGEN BioTECH) in the dark at room temperature for 30–60 min. Subsequently, cell cycle analysis was performed using the BD FACSVerse flow cytometer and Modfit LT software.

### Quantitative Real‐Time PCR

Total RNA from cell sampels was isolated using TRIzol reagent and reversed to cDNAs using HiScript III RT SuperMix for qPCR (+gDNA wiper) (Vazyme). Analysis of mRNA levels was performed using ChamQ SYBR qPCR Master Mix (Vazyme) by a LightCycler 96 System (Roche). Relative mRNA levels were calculated using the ∆∆Ct method by normalizing with GAPDH. Specific primers obtained from Tsingke Biotech for the target gene were as follows:

**Table:** 

GAPDH	Forward: 5’‐AACTTTGGCATTGTGGAAGG‐3’ Reverse: 5’‐ACACATTGGGGGTAGGAACA‐3’
RFC	Forward: 5’‐ACCATCGTCAAGACCATCATCAC‐3’ Reverse: 5’‐ ATGGACAGGATCAGGAAGTACAC‐3’
TAUT	Forward: 5’‐GGTGCGTTTCTCATACCGTATTT‐3’ Reverse: 5’‐ AGACATTCAGGAGGGACACAATT‐3’

### RNA‐seq and LC‐MS/MS

RNA‐seq library construction and sequencing were completed by TsingKe Biotech. LC‐MS/MS analysis of immunoprecipitated PDZK1 complexes was performed by Bioprofile Technology.

### Detection of ROS Fluorescence

Using the Reactive Oxygen Species Assay Kit (KeyGEN BioTECH), 1ml DCFH‐DA working solution was added per well of a six‐well plate after removing the cell culture medium and washing the cells twice with PBS. The cells were incubated at 37 °C for 20 min, washed three times with serum‐free culture medium. Fluorescence was measured on an inverted fluorescence microscope. For flow‐cytometric analysis, harvested cells were incubated with 10 µm DCFH‐DA at 37 °C for 20 min, washed three times, resuspended in serum‐free medium, and analyzed for fluorescence intensity on a BD FACSVerse flow cytometer.

### Measurement of Mitochondrial Membrane Potential

Mitochondrial membrane potential (ΔΨm) was measured by the JC‐1 mitochondrial membrane potential assay kit (KeyGEN BioTECH). Briefly, cells were seeded in a confocal dish, and after treatment, the detection of ΔΨm was performed following the protocol of JC‐1 kit. The fluorescence of JC‐1 was observed under a FluoView FV1000 confocal microscope (Olympus).

### Detection of Autophagosomes

According to the manufacturer's protocol, autophagosomes in cells were visualized by monodansylcadaverine (MDC) staining using the Autophagy Detection Kit (KeyGEN BioTECH).

### T‐AOC Assay

The cultured cells were digested with trypsin and centrifuged at 1500 rpm for 10 min. The supernatant was discarded, and the cell pellet was retained. To each tube, 0.5 mL of normal saline was added to prepare a cell suspension with a concentration of 10^7^ ml^−1^. Subsequently, the cell suspension was sonicated using an ultrasonic homogenizer. The reaction system was configured according to the instructions of the T‐AOC Detection Kit (KeyGEN BioTECH), and the experiment was carried out. Finally, the T‐AOC was analyzed by measuring the absorbance of each tube and calculating based on the formula provided in the instructions. At 37° C, an increase of 0.01 in the absorbance value of the reaction system per minute per milliliter of sample is defined as one unit of total antioxidant capacity (U).

### Ultra‐Wide‐Field Retinal Images Acquisition and Analysis

Ultra‐wide‐field swept‐source optical coherence tomography angiography (UWF‐SS‐OCTA, TowardPi Medical Technology Co., Ltd., Beijing, China) was performed using a 12 × 12 mm^2^ scan pattern centred around the optic nerve head (ONH) to collect OCT or vascular images of the retina. The following layers and parameters were automatically measured and analysed using the default software of the device, and any errors were manually corrected if necessary: the volume and average thickness of retinal nerve fiber layer (RNFL), inner nuclear layer (INL) and outer nuclear layer (ONL); the vessel flow density of the superficial capillary plexus layer (SCP, from the inner limiting membrane to the inner plexiform layer (IPL)) and the deep capillary plexus layer (DCP, from the IPL to the outer plexiform layer).

Wide‐angle color fundus photography and autofluorescence images were acquired using the Optos 200 Tx (Optos PLC, Dunfermline, United Kingdom).

### Electroretinography

After overnight dark adaptation, mice were anesthetized (65 mg kg^−1^ pentobarbital sodium), the cornea was anesthetized (1% proparacaine hydrochloride), and the pupils were dilated. Mice were placed on a temperature‐regulated heating pad throughout each recording session.

The flash electroretinography (FERG) items, including dark‐adaptation 3.0 response and dark‐adaptation 3.0 oscillatory potential response, were evaluated using the Visual Electrophysiology System (GT‐2008V, GOTEC, China).

### Histology and Immunofluorescence

Mice were euthanized, and eyeballs were removed and fixed in FAS Eye fixative (Servicebio) for 24h. Tissue sections of 4 µm were prepared as described previously.^[^ ^44^ ^]^ Before HE staining, 0.25g mlsampels decolorizing solution prepared from disinfection tablets (Lionser) in PBS was added to the tissues until RPE became colorless. HE Stain Kit (Solarbio) was subsequently used to perform the following the manufacturer's instructions.

The tissue immunofluorescence procedure followed the methods described previously.^[^ ^44^ ^]^ With the exception that antigen retrieval was performed by immersing the slides in Tris‐EDTA buffer (pH 9.0) at 65 °C for 6h.

For cell immunofluorescence staining, the cells were washed with phosphate‐buffered saline (PBS) and fixed with 4% paraformaldehyde for 20 min after treatment. Then, cells were solubilised with 0.4% Triton X‐100 at room temperature for 30 min. After washing three times with PBS, the cells were incubated overnight at 4 °C with primary antibodies, followed by incubation with corresponding second antibodies. Images were captured under a fluorescence microscope.

### Blood‐Retinal Barrier Leakage

Mice were injected with FITC‐BSA (80 mg µl^−1^, Solarbio) via the tail vein. After 15 min, the mice were sacrificed, and eyeballs were enucleated and immediately fixed in 4% paraformaldehyde for 20 min. Retinal whole mounts were then prepared and examined under a fluorescence microscope.

### Statistical Analysis

All experimental data are expressed as the mean ± SD of at least three independent experiments. Statistical analyses were performed using a two‐tailed Student's *t*‐test or one‐way ANOVA followed by Tukey's test in Prism 9 (GraphPad, Boston, MA, USA). *P* < 0.05 was considered significant.

## Conflict of Interest

The authors declare no conflict of interest.

## Supporting information



Supporting Information

## Data Availability

The data supporting this study are openly available in the NCBI GEO database under accession numbers GSE304690 and GSE304545. The data supporting this study are openly available at:
GSE304690:
https://urldefense.com/v3/__http://www.ncbi.nlm.nih.gov/geo/query/acc.cgi?acc=GSE304690__;!!N11eV2iwtfs!pEQeT2NwqiKleYkNPGUeEgoOjX4g-QxnVzpjXxRvzUfv02QYGFOkrV1-X2sWfmRyjeNBUPFjjV5hXUJqPZKu$
GSE304545:
https://urldefense.com/v3/__http://www.ncbi.nlm.nih.gov/geo/query/acc.cgi?acc=GSE304545__;!!N11eV2iwtfs!pEQeT2NwqiKleYkNPGUeEgoOjX4g-QxnVzpjXxRvzUfv02QYGFOkrV1-X2sWfmRyjeNBUPFjjV5hXdi0TLlh$ All other supporting data are available upon reasonable request.
