# Supplementary material for: PDZK1 Protects Against RPE Senescence by Targeting the 14‐3‐3ε‐mTOR Axis to Attenuate Early Diabetic Retinopathy
Source: Adv Sci (Weinh). 2025 Aug 25;12(43):e11288. doi: 10.1002/advs.202511288 (PMC12631858; doi:10.1002/advs.202511288)
Supplement: Supplementary file 1 — Supporting Information [file ADVS-12-e11288-s001.docx]

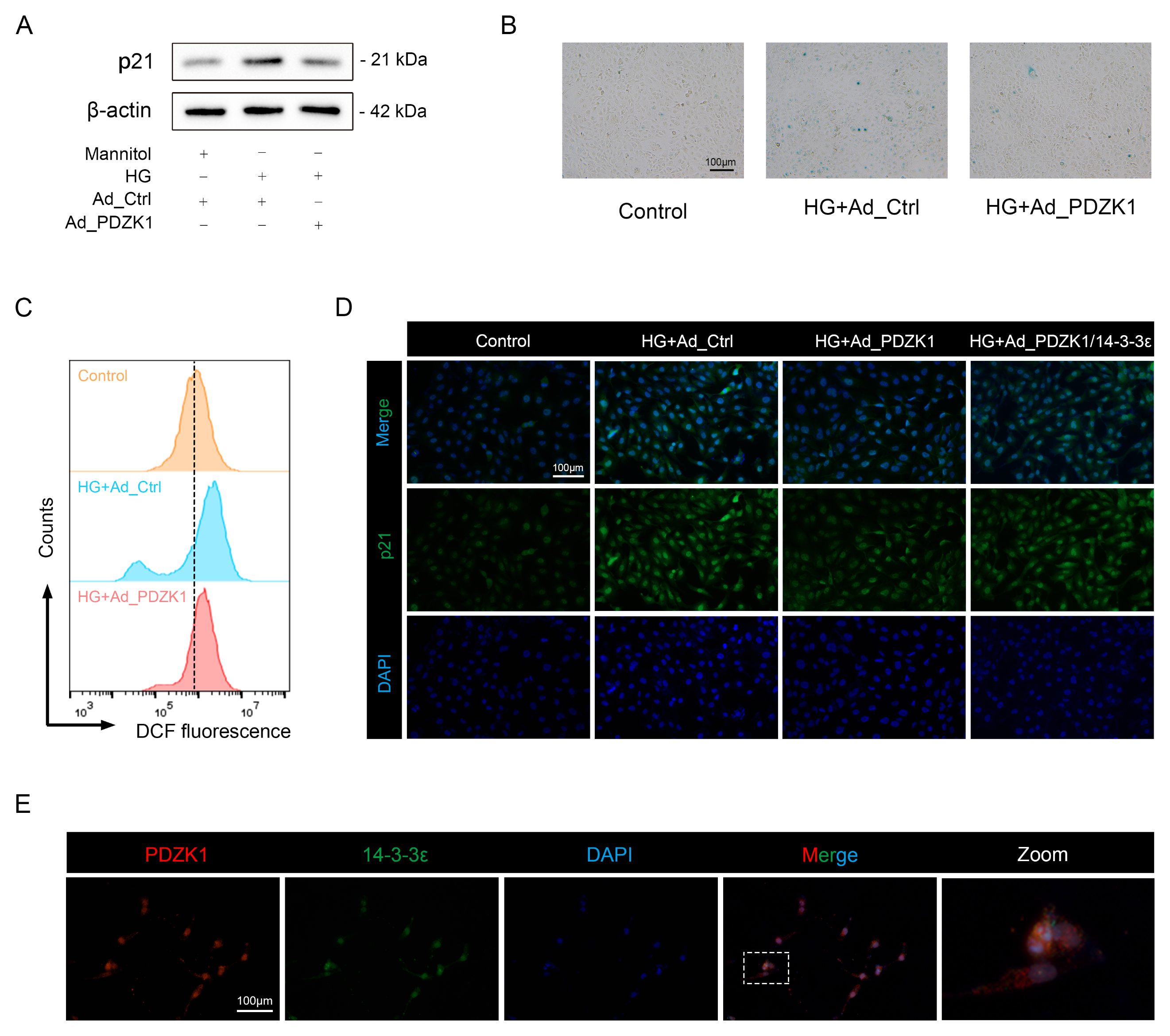


**Figure S1.** (A) Western blot analysis of p21 protein expression levels in human primary RPE cells. (B) Representative images of SA-β-gal staining in human primary RPE cells. (C) Flow-cytometric quantification of intracellular ROS using the DCFH-DA probe in human primary RPE cells. (D) Representative images of immunofluorescence assessment of p21 in human primary RPE cells. (E) Immunofluorescence colocalization of PDZK1 and 14-3-3ε in human primary RPE cells. All experiments were performed with a minimum of three biological replicates.
